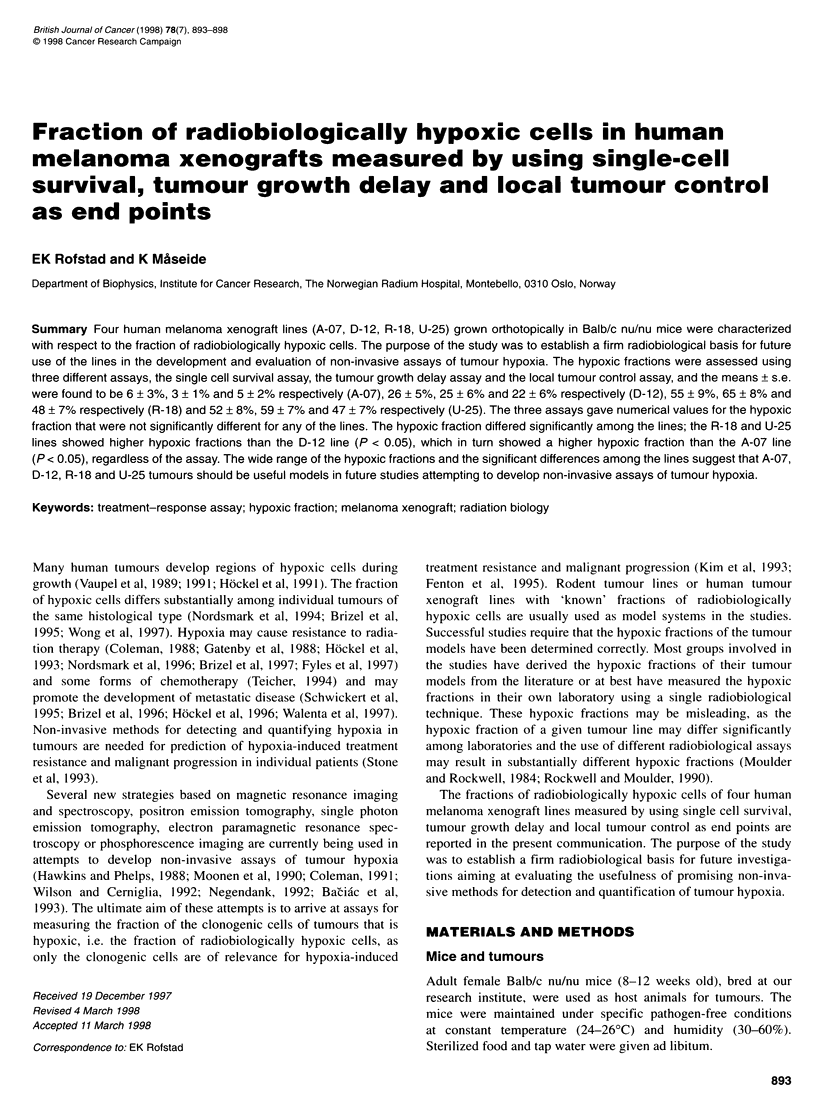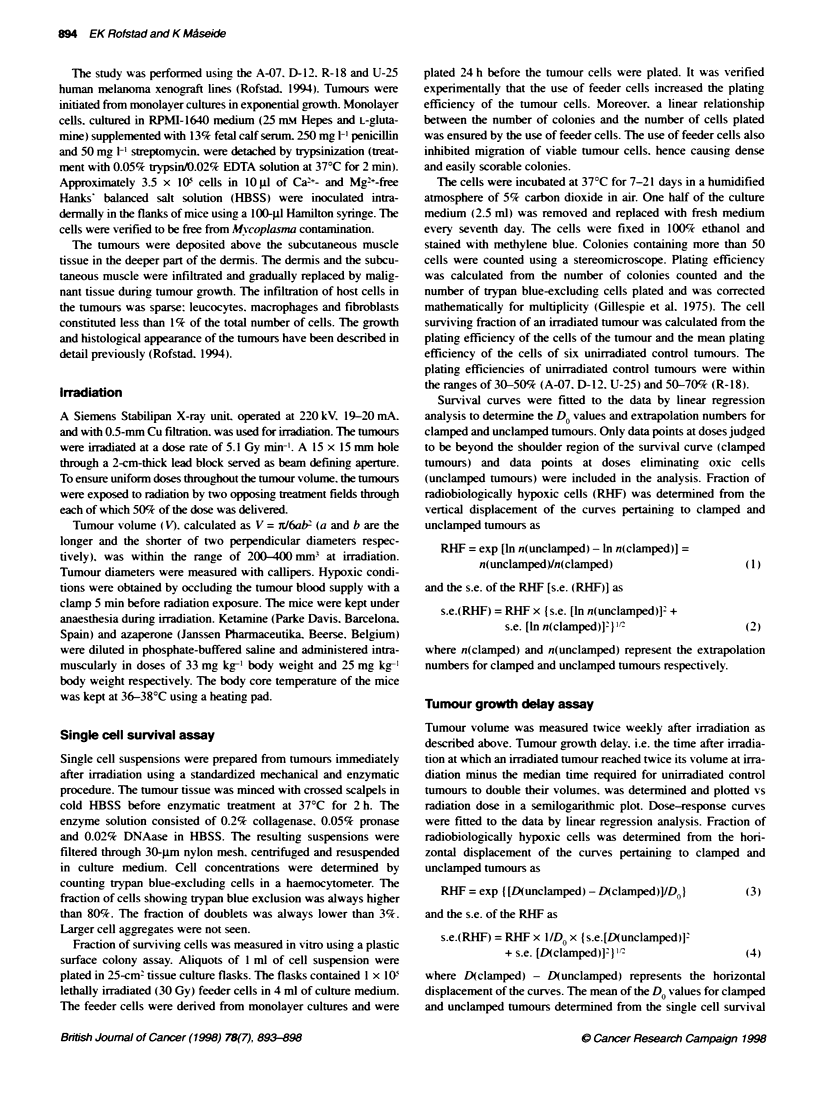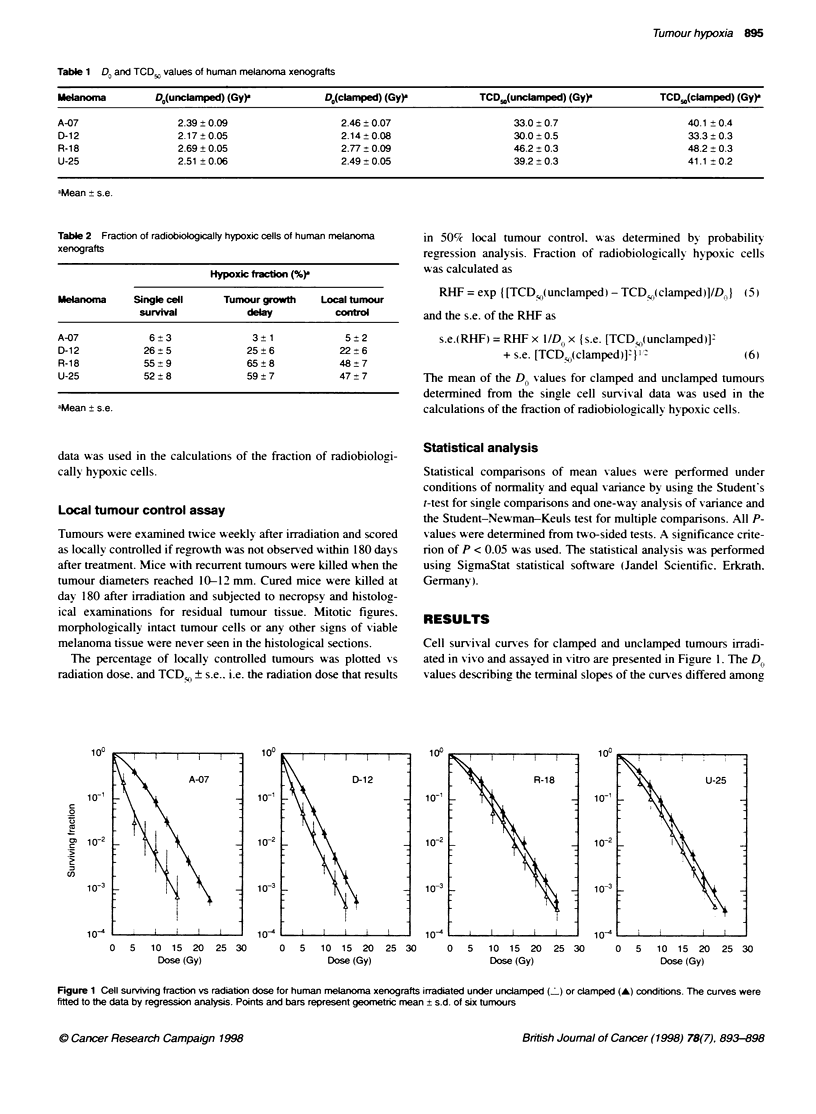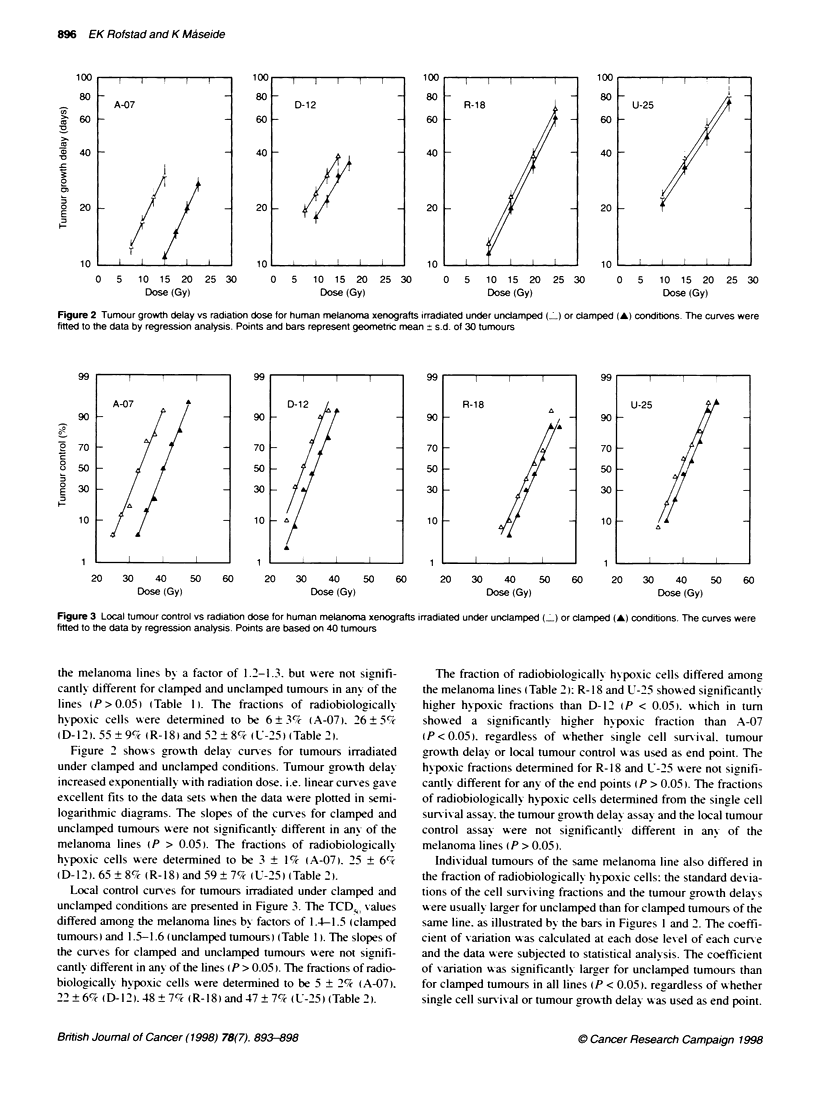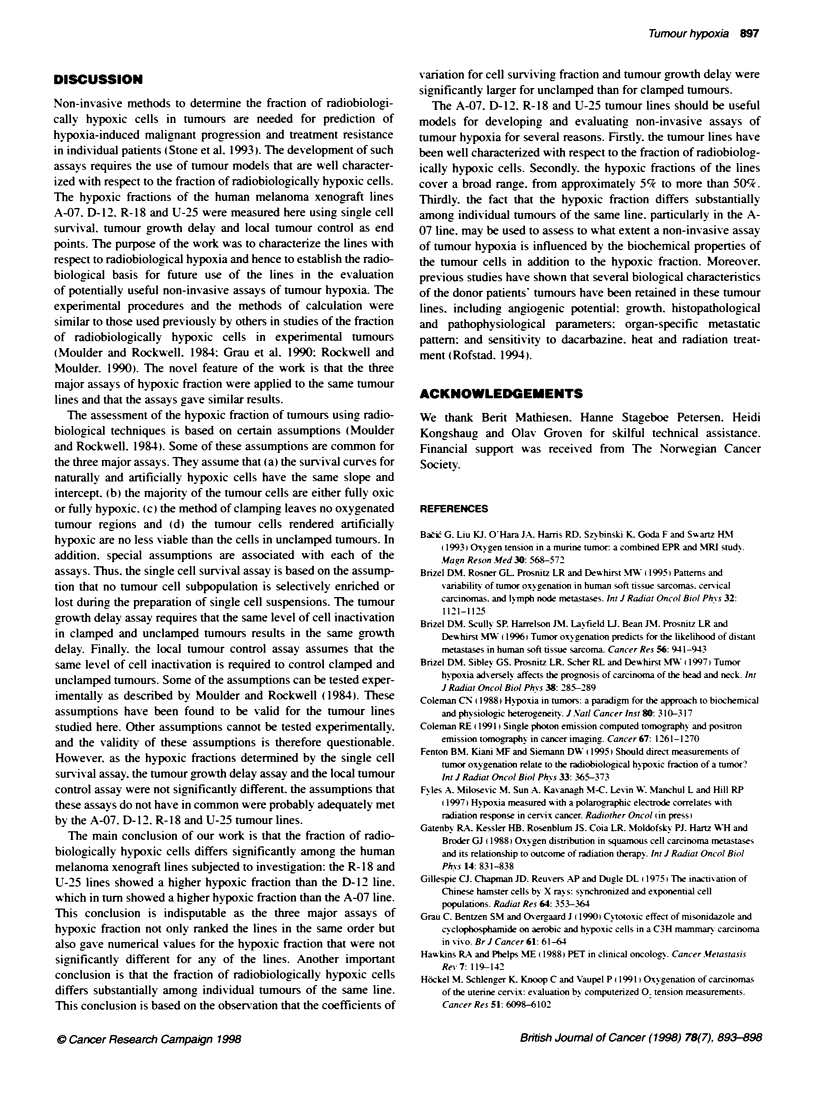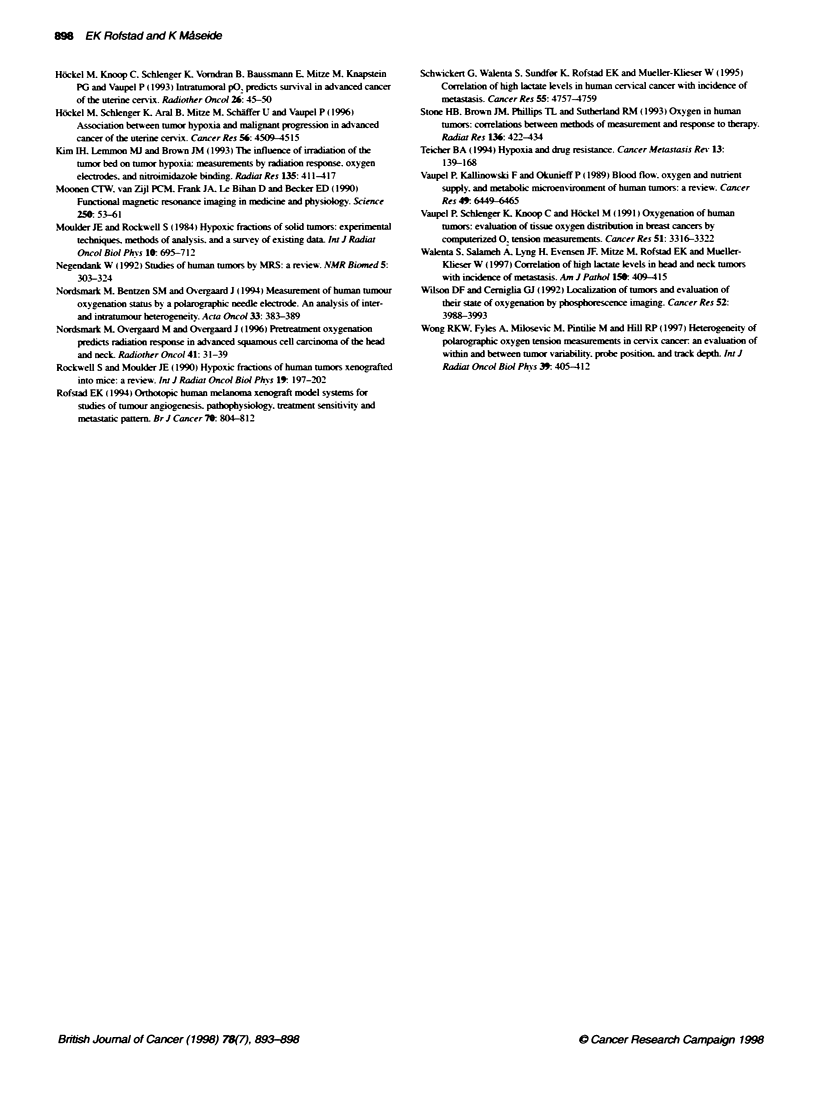# Fraction of radiobiologically hypoxic cells in human melanoma xenografts measured by using single-cell survival, tumour growth delay and local tumour control as end points.

**DOI:** 10.1038/bjc.1998.598

**Published:** 1998-10

**Authors:** E. K. Rofstad, K. Måseide

**Affiliations:** Department of Biophysics, Institute for Cancer Research, The Norwegian Radium Hospital, Montebello, Oslo.

## Abstract

Four human melanoma xenograft lines (A-07, D-12, R-18, U-25) grown orthotopically in Balb/c nu/nu mice were characterized with respect to the fraction of radiobiologically hypoxic cells. The purpose of the study was to establish a firm radiobiological basis for future use of the lines in the development and evaluation of non-invasive assays of tumour hypoxia. The hypoxic fractions were assessed using three different assays, the single cell survival assay, the tumour growth delay assay and the local tumour control assay, and the means +/- s.e. were found to be 6 +/- 3%, 3 +/- 1% and 5 +/- 2% respectively (A-07), 26 +/- 5%, 25 +/- 6% and 22 +/- 6% respectively (D-12), 55 +/- 9%, 65 +/- 8% and 48 +/- 7% respectively (R-18) and 52 +/- 8%, 59 +/- 7% and 47 +/- 7% respectively (U-25). The three assays gave numerical values for the hypoxic fraction that were not significantly different for any of the lines. The hypoxic fraction differed significantly among the lines; the R-18 and U-25 lines showed higher hypoxic fractions than the D-12 line (P < 0.05), which in turn showed a higher hypoxic fraction than the A-07 line (P < 0.05), regardless of the assay. The wide range of the hypoxic fractions and the significant differences among the lines suggest that A-07, D-12. R-18 and U-25 tumours should be useful models in future studies attempting to develop non-invasive assays of tumour hypoxia.


British Journal of Cancer (1998) 78(7), 893–898
© 1998 Cancer Research Campaign

### Fraction of radiobiologically hypoxic cells in human melanoma xenografts measured by using single-cell survival, tumour growth delay and local tumour control as end points

**EK Rofstad and K Måseide**

Department of Biophysics, Institute for Cancer Research, The Norwegian Radium Hospital, Montebello, 0310 Oslo, Norway

**Summary** Four human melanoma xenograft lines (A-07, D-12, R-18, U-25) grown orthotopically in Balb/c nu/nu mice were characterized with respect to the fraction of radiobiologically hypoxic cells. The purpose of the study was to establish a firm radiobiological basis for future use of the lines in the development and evaluation of non-invasive assays of tumour hypoxia. The hypoxic fractions were assessed using three different assays, the single cell survival assay, the tumour growth delay assay and the local tumour control assay, and the means ± s.e. were found to be 6 ± 3%, 3 ± 1% and 5 ± 2% respectively (A-07), 26 ± 5%, 25 ± 6% and 22 ± 6% respectively (D-12), 55 ± 9%, 65 ± 8% and 48 ± 7% respectively (R-18) and 52 ± 8%, 59 ± 7% and 47 ± 7% respectively (U-25). The three assays gave numerical values for the hypoxic fraction that were not significantly different for any of the lines. The hypoxic fraction differed significantly among the lines; the R-18 and U-25 lines showed higher hypoxic fractions than the D-12 line ($P < 0.05$), which in turn showed a higher hypoxic fraction than the A-07 line ($P < 0.05$), regardless of the assay. The wide range of the hypoxic fractions and the significant differences among the lines suggest that A-07, D-12, R-18 and U-25 tumours should be useful models in future studies attempting to develop non-invasive assays of tumour hypoxia.

**Keywords:** treatment–response assay; hypoxic fraction; melanoma xenograft; radiation biology

Many human tumours develop regions of hypoxic cells during growth (Vaupel et al, 1989; 1991; Höckel et al, 1991). The fraction of hypoxic cells differs substantially among individual tumours of the same histological type (Nordsmark et al, 1994; Brizel et al, 1995; Wong et al, 1997). Hypoxia may cause resistance to radiation therapy (Coleman, 1988; Gatenby et al, 1988; Höckel et al, 1993; Nordsmark et al, 1996; Brizel et al, 1997; Fyles et al, 1997) and some forms of chemotherapy (Teicher, 1994) and may promote the development of metastatic disease (Schwickert et al, 1995; Brizel et al, 1996; Höckel et al, 1996; Walenta et al, 1997). Non-invasive methods for detecting and quantifying hypoxia in tumours are needed for prediction of hypoxia-induced treatment resistance and malignant progression in individual patients (Stone et al, 1993).

Several new strategies based on magnetic resonance imaging and spectroscopy, positron emission tomography, single photon emission tomography, electron paramagnetic resonance spectroscopy or phosphorescence imaging are currently being used in attempts to develop non-invasive assays of tumour hypoxia (Hawkins and Phelps, 1988; Moonen et al, 1990; Coleman, 1991; Wilson and Cerniglia, 1992; Negendank, 1992; Bačiác et al, 1993). The ultimate aim of these attempts is to arrive at assays for measuring the fraction of the clonogenic cells of tumours that is hypoxic, i.e. the fraction of radiobiologically hypoxic cells, as only the clonogenic cells are of relevance for hypoxia-induced treatment resistance and malignant progression (Kim et al, 1993; Fenton et al, 1995). Rodent tumour lines or human tumour xenograft lines with 'known' fractions of radiobiologically hypoxic cells are usually used as model systems in the studies. Successful studies require that the hypoxic fractions of the tumour models have been determined correctly. Most groups involved in the studies have derived the hypoxic fractions of their tumour models from the literature or at best have measured the hypoxic fractions in their own laboratory using a single radiobiological technique. These hypoxic fractions may be misleading, as the hypoxic fraction of a given tumour line may differ significantly among laboratories and the use of different radiobiological assays may result in substantially different hypoxic fractions (Moulder and Rockwell, 1984; Rockwell and Moulder, 1990).

The fractions of radiobiologically hypoxic cells of four human melanoma xenograft lines measured by using single cell survival, tumour growth delay and local tumour control as end points are reported in the present communication. The purpose of the study was to establish a firm radiobiological basis for future investigations aiming at evaluating the usefulness of promising non-invasive methods for detection and quantification of tumour hypoxia.

### MATERIALS AND METHODS

### Mice and tumours

Adult female Balb/c nu/nu mice (8–12 weeks old), bred at our research institute, were used as host animals for tumours. The mice were maintained under specific pathogen-free conditions at constant temperature (24–26°C) and humidity (30–60%). Sterilized food and tap water were given ad libitum.

Received 19 December 1997
Revised 4 March 1998
Accepted 11 March 1998

Correspondence to: EK Rofstad

The study was performed using the A-07, D-12, R-18 and U-25 human melanoma xenograft lines (Rofstad, 1994). Tumours were initiated from monolayer cultures in exponential growth. Monolayer cells, cultured in RPMI-1640 medium (25 mM Hepes and L-glutamine) supplemented with 13% fetal calf serum, 250 mg $l^{-1}$ penicillin and 50 mg $l^{-1}$ streptomycin, were detached by trypsinization (treatment with 0.05% trypsin/0.02% EDTA solution at 37°C for 2 min). Approximately 3.5 × $10^5$ cells in 10 µl of $Ca^{2+}$- and $Mg^{2+}$-free Hanks' balanced salt solution (HBSS) were inoculated intradermally in the flanks of mice using a 100-µl Hamilton syringe. The cells were verified to be free from *Mycoplasma* contamination.

The tumours were deposited above the subcutaneous muscle tissue in the deeper part of the dermis. The dermis and the subcutaneous muscle were infiltrated and gradually replaced by malignant tissue during tumour growth. The infiltration of host cells in the tumours was sparse: leucocytes, macrophages and fibroblasts constituted less than 1% of the total number of cells. The growth and histological appearance of the tumours have been described in detail previously (Rofstad, 1994).

### Irradiation

A Siemens Stabilipan X-ray unit, operated at 220 kV, 19–20 mA, and with 0.5-mm Cu filtration, was used for irradiation. The tumours were irradiated at a dose rate of 5.1 Gy $min^{-1}$. A 15 × 15 mm hole through a 2-cm-thick lead block served as beam defining aperture. To ensure uniform doses throughout the tumour volume, the tumours were exposed to radiation by two opposing treatment fields through each of which 50% of the dose was delivered.

Tumour volume ($V$), calculated as $V = \pi/6ab^2$ ($a$ and $b$ are the longer and the shorter of two perpendicular diameters respectively), was within the range of 200–400 $mm^3$ at irradiation. Tumour diameters were measured with callipers. Hypoxic conditions were obtained by occluding the tumour blood supply with a clamp 5 min before radiation exposure. The mice were kept under anaesthesia during irradiation. Ketamine (Parke Davis, Barcelona, Spain) and azaperone (Janssen Pharmaceutika, Beerse, Belgium) were diluted in phosphate-buffered saline and administered intramuscularly in doses of 33 mg $kg^{-1}$ body weight and 25 mg $kg^{-1}$ body weight respectively. The body core temperature of the mice was kept at 36–38°C using a heating pad.

### Single cell survival assay

Single cell suspensions were prepared from tumours immediately after irradiation using a standardized mechanical and enzymatic procedure. The tumour tissue was minced with crossed scalpels in cold HBSS before enzymatic treatment at 37°C for 2 h. The enzyme solution consisted of 0.2% collagenase, 0.05% pronase and 0.02% DNAase in HBSS. The resulting suspensions were filtered through 30-µm nylon mesh, centrifuged and resuspended in culture medium. Cell concentrations were determined by counting trypan blue-excluding cells in a haemocytometer. The fraction of cells showing trypan blue exclusion was always higher than 80%. The fraction of doublets was always lower than 3%. Larger cell aggregates were not seen.

Fraction of surviving cells was measured in vitro using a plastic surface colony assay. Aliquots of 1 ml of cell suspension were plated in 25-$cm^2$ tissue culture flasks. The flasks contained 1 × $10^5$ lethally irradiated (30 Gy) feeder cells in 4 ml of culture medium. The feeder cells were derived from monolayer cultures and were plated 24 h before the tumour cells were plated. It was verified experimentally that the use of feeder cells increased the plating efficiency of the tumour cells. Moreover, a linear relationship between the number of colonies and the number of cells plated was ensured by the use of feeder cells. The use of feeder cells also inhibited migration of viable tumour cells, hence causing dense and easily scorable colonies.

The cells were incubated at 37°C for 7–21 days in a humidified atmosphere of 5% carbon dioxide in air. One half of the culture medium (2.5 ml) was removed and replaced with fresh medium every seventh day. The cells were fixed in 100% ethanol and stained with methylene blue. Colonies containing more than 50 cells were counted using a stereomicroscope. Plating efficiency was calculated from the number of colonies counted and the number of trypan blue-excluding cells plated and was corrected mathematically for multiplicity (Gillespie et al, 1975). The cell surviving fraction of an irradiated tumour was calculated from the plating efficiency of the cells of the tumour and the mean plating efficiency of the cells of six unirradiated control tumours. The plating efficiencies of unirradiated control tumours were within the ranges of 30–50% (A-07, D-12, U-25) and 50–70% (R-18).

Survival curves were fitted to the data by linear regression analysis to determine the $D_0$ values and extrapolation numbers for clamped and unclamped tumours. Only data points at doses judged to be beyond the shoulder region of the survival curve (clamped tumours) and data points at doses eliminating oxic cells (unclamped tumours) were included in the analysis. Fraction of radiobiologically hypoxic cells (RHF) was determined from the vertical displacement of the curves pertaining to clamped and unclamped tumours as

$$\mathrm{RHF} = \exp\,[\ln n(\mathrm{unclamped}) - \ln n(\mathrm{clamped})] = n(\mathrm{unclamped})/n(\mathrm{clamped}) \qquad (1)$$

and the s.e. of the RHF [s.e. (RHF)] as

$$\mathrm{s.e.(RHF)} = \mathrm{RHF} \times \{\mathrm{s.e.}\,[\ln n(\mathrm{unclamped})]^2 + \mathrm{s.e.}\,[\ln n(\mathrm{clamped})]^2\}^{1/2} \qquad (2)$$

where $n$(clamped) and $n$(unclamped) represent the extrapolation numbers for clamped and unclamped tumours respectively.

### Tumour growth delay assay

Tumour volume was measured twice weekly after irradiation as described above. Tumour growth delay, i.e. the time after irradiation at which an irradiated tumour reached twice its volume at irradiation minus the median time required for unirradiated control tumours to double their volumes, was determined and plotted vs radiation dose in a semilogarithmic plot. Dose–response curves were fitted to the data by linear regression analysis. Fraction of radiobiologically hypoxic cells was determined from the horizontal displacement of the curves pertaining to clamped and unclamped tumours as

$$\mathrm{RHF} = \exp\,\{[D(\mathrm{unclamped}) - D(\mathrm{clamped})]/D_0\} \qquad (3)$$

and the s.e. of the RHF as

$$\mathrm{s.e.(RHF)} = \mathrm{RHF} \times 1/D_0 \times \{\mathrm{s.e.}[D(\mathrm{unclamped})]^2 + \mathrm{s.e.}\,[D(\mathrm{clamped})]^2\}^{1/2} \qquad (4)$$

where $D$(clamped) – $D$(unclamped) represents the horizontal displacement of the curves. The mean of the $D_0$ values for clamped and unclamped tumours determined from the single cell survival

© Cancer Research Campaign 1998

**Table 1** $D_0$ and $TCD_{50}$ values of human melanoma xenografts

| Melanoma | $D_0$(unclamped) (Gy)[a] | $D_0$(clamped) (Gy)[a] | $TCD_{50}$(unclamped) (Gy)[a] | $TCD_{50}$(clamped) (Gy)[a] |
|---|---|---|---|---|
| A-07 | 2.39 ± 0.09 | 2.46 ± 0.07 | 33.0 ± 0.7 | 40.1 ± 0.4 |
| D-12 | 2.17 ± 0.05 | 2.14 ± 0.08 | 30.0 ± 0.5 | 33.3 ± 0.3 |
| R-18 | 2.69 ± 0.05 | 2.77 ± 0.09 | 46.2 ± 0.3 | 48.2 ± 0.3 |
| U-25 | 2.51 ± 0.06 | 2.49 ± 0.05 | 39.2 ± 0.3 | 41.1 ± 0.2 |

[a]Mean ± s.e.

**Table 2** Fraction of radiobiologically hypoxic cells of human melanoma xenografts

| | Hypoxic fraction (%)[a] | | |
|---|---|---|---|
| Melanoma | Single cell survival | Tumour growth delay | Local tumour control |
| A-07 | 6 ± 3 | 3 ± 1 | 5 ± 2 |
| D-12 | 26 ± 5 | 25 ± 6 | 22 ± 6 |
| R-18 | 55 ± 9 | 65 ± 8 | 48 ± 7 |
| U-25 | 52 ± 8 | 59 ± 7 | 47 ± 7 |

[a]Mean ± s.e.

data was used in the calculations of the fraction of radiobiologically hypoxic cells.

### Local tumour control assay

Tumours were examined twice weekly after irradiation and scored as locally controlled if regrowth was not observed within 180 days after treatment. Mice with recurrent tumours were killed when the tumour diameters reached 10–12 mm. Cured mice were killed at day 180 after irradiation and subjected to necropsy and histological examinations for residual tumour tissue. Mitotic figures, morphologically intact tumour cells or any other signs of viable melanoma tissue were never seen in the histological sections.

The percentage of locally controlled tumours was plotted vs radiation dose, and $TCD_{50}$ ± s.e., i.e. the radiation dose that results in 50% local tumour control, was determined by probability regression analysis. Fraction of radiobiologically hypoxic cells was calculated as

$$RHF = \exp\{[TCD_{50}(\text{unclamped}) - TCD_{50}(\text{clamped})]/D_0\} \quad (5)$$

and the s.e. of the RHF as

$$\text{s.e.}(RHF) = RHF \times 1/D_0 \times \{\text{s.e.}\,[TCD_{50}(\text{unclamped})]^2 + \text{s.e.}\,[TCD_{50}(\text{clamped})]^2\}^{1/2} \quad (6)$$

The mean of the $D_0$ values for clamped and unclamped tumours determined from the single cell survival data was used in the calculations of the fraction of radiobiologically hypoxic cells.

### Statistical analysis

Statistical comparisons of mean values were performed under conditions of normality and equal variance by using the Student's $t$-test for single comparisons and one-way analysis of variance and the Student–Newman–Keuls test for multiple comparisons. All $P$-values were determined from two-sided tests. A significance criterion of $P < 0.05$ was used. The statistical analysis was performed using SigmaStat statistical software (Jandel Scientific, Erkrath, Germany).

### RESULTS

Cell survival curves for clamped and unclamped tumours irradiated in vivo and assayed in vitro are presented in Figure 1. The $D_0$ values describing the terminal slopes of the curves differed among

**Figure 1** Cell surviving fraction vs radiation dose for human melanoma xenografts irradiated under unclamped (△) or clamped (▲) conditions. The curves were fitted to the data by regression analysis. Points and bars represent geometric mean ± s.d. of six tumours

**Figure 2** Tumour growth delay vs radiation dose for human melanoma xenografts irradiated under unclamped (△) or clamped (▲) conditions. The curves were fitted to the data by regression analysis. Points and bars represent geometric mean ± s.d. of 30 tumours

**Figure 3** Local tumour control vs radiation dose for human melanoma xenografts irradiated under unclamped (△) or clamped (▲) conditions. The curves were fitted to the data by regression analysis. Points are based on 40 tumours the melanoma lines by a factor of 1.2–1.3, but were not significantly different for clamped and unclamped tumours in any of the lines ($P > 0.05$) (Table 1). The fractions of radiobiologically hypoxic cells were determined to be 6 ± 3% (A-07), 26 ± 5% (D-12), 55 ± 9% (R-18) and 52 ± 8% (U-25) (Table 2).

Figure 2 shows growth delay curves for tumours irradiated under clamped and unclamped conditions. Tumour growth delay increased exponentially with radiation dose, i.e. linear curves gave excellent fits to the data sets when the data were plotted in semi-logarithmic diagrams. The slopes of the curves for clamped and unclamped tumours were not significantly different in any of the melanoma lines ($P > 0.05$). The fractions of radiobiologically hypoxic cells were determined to be 3 ± 1% (A-07), 25 ± 6% (D-12), 65 ± 8% (R-18) and 59 ± 7% (U-25) (Table 2).

Local control curves for tumours irradiated under clamped and unclamped conditions are presented in Figure 3. The $TCD_{50}$ values differed among the melanoma lines by factors of 1.4–1.5 (clamped tumours) and 1.5–1.6 (unclamped tumours) (Table 1). The slopes of the curves for clamped and unclamped tumours were not significantly different in any of the lines ($P > 0.05$). The fractions of radiobiologically hypoxic cells were determined to be 5 ± 2% (A-07), 22 ± 6% (D-12), 48 ± 7% (R-18) and 47 ± 7% (U-25) (Table 2).

The fraction of radiobiologically hypoxic cells differed among the melanoma lines (Table 2): R-18 and U-25 showed significantly higher hypoxic fractions than D-12 ($P < 0.05$), which in turn showed a significantly higher hypoxic fraction than A-07 ($P < 0.05$), regardless of whether single cell survival, tumour growth delay or local tumour control was used as end point. The hypoxic fractions determined for R-18 and U-25 were not significantly different for any of the end points ($P > 0.05$). The fractions of radiobiologically hypoxic cells determined from the single cell survival assay, the tumour growth delay assay and the local tumour control assay were not significantly different in any of the melanoma lines ($P > 0.05$).

Individual tumours of the same melanoma line also differed in the fraction of radiobiologically hypoxic cells: the standard deviations of the cell surviving fractions and the tumour growth delays were usually larger for unclamped than for clamped tumours of the same line, as illustrated by the bars in Figures 1 and 2. The coefficient of variation was calculated at each dose level of each curve and the data were subjected to statistical analysis. The coefficient of variation was significantly larger for unclamped tumours than for clamped tumours in all lines ($P < 0.05$), regardless of whether single cell survival or tumour growth delay was used as end point.

© Cancer Research Campaign 1998

### DISCUSSION

Non-invasive methods to determine the fraction of radiobiologically hypoxic cells in tumours are needed for prediction of hypoxia-induced malignant progression and treatment resistance in individual patients (Stone et al. 1993). The development of such assays requires the use of tumour models that are well characterized with respect to the fraction of radiobiologically hypoxic cells. The hypoxic fractions of the human melanoma xenograft lines A-07, D-12, R-18 and U-25 were measured here using single cell survival, tumour growth delay and local tumour control as end points. The purpose of the work was to characterize the lines with respect to radiobiological hypoxia and hence to establish the radiobiological basis for future use of the lines in the evaluation of potentially useful non-invasive assays of tumour hypoxia. The experimental procedures and the methods of calculation were similar to those used previously by others in studies of the fraction of radiobiologically hypoxic cells in experimental tumours (Moulder and Rockwell, 1984; Grau et al, 1990; Rockwell and Moulder, 1990). The novel feature of the work is that the three major assays of hypoxic fraction were applied to the same tumour lines and that the assays gave similar results.

The assessment of the hypoxic fraction of tumours using radiobiological techniques is based on certain assumptions (Moulder and Rockwell, 1984). Some of these assumptions are common for the three major assays. They assume that (a) the survival curves for naturally and artificially hypoxic cells have the same slope and intercept, (b) the majority of the tumour cells are either fully oxic or fully hypoxic, (c) the method of clamping leaves no oxygenated tumour regions and (d) the tumour cells rendered artificially hypoxic are no less viable than the cells in unclamped tumours. In addition, special assumptions are associated with each of the assays. Thus, the single cell survival assay is based on the assumption that no tumour cell subpopulation is selectively enriched or lost during the preparation of single cell suspensions. The tumour growth delay assay requires that the same level of cell inactivation in clamped and unclamped tumours results in the same growth delay. Finally, the local tumour control assay assumes that the same level of cell inactivation is required to control clamped and unclamped tumours. Some of the assumptions can be tested experimentally as described by Moulder and Rockwell (1984). These assumptions have been found to be valid for the tumour lines studied here. Other assumptions cannot be tested experimentally, and the validity of these assumptions is therefore questionable. However, as the hypoxic fractions determined by the single cell survival assay, the tumour growth delay assay and the local tumour control assay were not significantly different, the assumptions that these assays do not have in common were probably adequately met by the A-07, D-12, R-18 and U-25 tumour lines.

The main conclusion of our work is that the fraction of radiobiologically hypoxic cells differs significantly among the human melanoma xenograft lines subjected to investigation: the R-18 and U-25 lines showed a higher hypoxic fraction than the D-12 line, which in turn showed a higher hypoxic fraction than the A-07 line. This conclusion is indisputable as the three major assays of hypoxic fraction not only ranked the lines in the same order but also gave numerical values for the hypoxic fraction that were not significantly different for any of the lines. Another important conclusion is that the fraction of radiobiologically hypoxic cells differs substantially among individual tumours of the same line. This conclusion is based on the observation that the coefficients of variation for cell surviving fraction and tumour growth delay were significantly larger for unclamped than for clamped tumours.

The A-07, D-12, R-18 and U-25 tumour lines should be useful models for developing and evaluating non-invasive assays of tumour hypoxia for several reasons. Firstly, the tumour lines have been well characterized with respect to the fraction of radiobiologically hypoxic cells. Secondly, the hypoxic fractions of the lines cover a broad range, from approximately 5% to more than 50%. Thirdly, the fact that the hypoxic fraction differs substantially among individual tumours of the same line, particularly in the A-07 line, may be used to assess to what extent a non-invasive assay of tumour hypoxia is influenced by the biochemical properties of the tumour cells in addition to the hypoxic fraction. Moreover, previous studies have shown that several biological characteristics of the donor patients' tumours have been retained in these tumour lines, including angiogenic potential; growth, histopathological and pathophysiological parameters; organ-specific metastatic pattern; and sensitivity to dacarbazine, heat and radiation treatment (Rofstad, 1994).

### ACKNOWLEDGEMENTS

We thank Berit Mathiesen, Hanne Stageboe Petersen, Heidi Kongshaug and Olav Groven for skilful technical assistance. Financial support was received from The Norwegian Cancer Society.

### REFERENCES

Bačić G, Liu KJ, O'Hara JA, Harris RD, Szybinski K, Goda F and Swartz HM (1993) Oxygen tension in a murine tumor: a combined EPR and MRI study. *Magn Reson Med* **30**: 568–572

Brizel DM, Rosner GL, Prosnitz LR and Dewhirst MW (1995) Patterns and variability of tumor oxygenation in human soft tissue sarcomas, cervical carcinomas, and lymph node metastases. *Int J Radiat Oncol Biol Phys* **32**: 1121–1125

Brizel DM, Scully SP, Harrelson JM, Layfield LJ, Bean JM, Prosnitz LR and Dewhirst MW (1996) Tumor oxygenation predicts for the likelihood of distant metastases in human soft tissue sarcoma. *Cancer Res* **56**: 941–943

Brizel DM, Sibley GS, Prosnitz LR, Scher RL and Dewhirst MW (1997) Tumor hypoxia adversely affects the prognosis of carcinoma of the head and neck. *Int J Radiat Oncol Biol Phys* **38**: 285–289

Coleman CN (1988) Hypoxia in tumors: a paradigm for the approach to biochemical and physiologic heterogeneity. *J Natl Cancer Inst* **80**: 310–317

Coleman RE (1991) Single photon emission computed tomography and positron emission tomography in cancer imaging. *Cancer* **67**: 1261–1270

Fenton BM, Kiani MF and Siemann DW (1995) Should direct measurements of tumor oxygenation relate to the radiobiological hypoxic fraction of a tumor? *Int J Radiat Oncol Biol Phys* **33**: 365–373

Fyles A, Milosevic M, Sun A, Kavanagh M-C, Levin W, Manchul L and Hill RP (1997) Hypoxia measured with a polarographic electrode correlates with radiation response in cervix cancer. *Radiother Oncol* (in press)

Gatenby RA, Kessler HB, Rosenblum JS, Coia LR, Moldofsky PJ, Hartz WH and Broder GJ (1988) Oxygen distribution in squamous cell carcinoma metastases and its relationship to outcome of radiation therapy. *Int J Radiat Oncol Biol Phys* **14**: 831–838

Gillespie CJ, Chapman JD, Reuvers AP and Dugle DL (1975) The inactivation of Chinese hamster cells by X rays: synchronized and exponential cell populations. *Radiat Res* **64**: 353–364

Grau C, Bentzen SM and Overgaard J (1990) Cytotoxic effect of misonidazole and cyclophosphamide on aerobic and hypoxic cells in a C3H mammary carcinoma in vivo. *Br J Cancer* **61**: 61–64

Hawkins RA and Phelps ME (1988) PET in clinical oncology. *Cancer Metastasis Rev* **7**: 119–142

Höckel M, Schlenger K, Knoop C and Vaupel P (1991) Oxygenation of carcinomas of the uterine cervix: evaluation by computerized $O_2$ tension measurements. *Cancer Res* **51**: 6098–6102

Höckel M, Knoop C, Schlenger K, Vorndran B, Baussmann E, Mitze M, Knapstein PG and Vaupel P (1993) Intratumoral $pO_2$ predicts survival in advanced cancer of the uterine cervix. *Radiother Oncol* **26**: 45–50

Höckel M, Schlenger K, Aral B, Mitze M, Schäffer U and Vaupel P (1996) Association between tumor hypoxia and malignant progression in advanced cancer of the uterine cervix. *Cancer Res* **56**: 4509–4515

Kim IH, Lemmon MJ and Brown JM (1993) The influence of irradiation of the tumor bed on tumor hypoxia: measurements by radiation response, oxygen electrodes, and nitroimidazole binding. *Radiat Res* **135**: 411–417

Moonen CTW, van Zijl PCM, Frank JA, Le Bihan D and Becker ED (1990) Functional magnetic resonance imaging in medicine and physiology. *Science* **250**: 53–61

Moulder JE and Rockwell S (1984) Hypoxic fractions of solid tumors: experimental techniques, methods of analysis, and a survey of existing data. *Int J Radiat Oncol Biol Phys* **10**: 695–712

Negendank W (1992) Studies of human tumors by MRS: a review. *NMR Biomed* **5**: 303–324

Nordsmark M, Bentzen SM and Overgaard J (1994) Measurement of human tumour oxygenation status by a polarographic needle electrode. An analysis of inter- and intratumour heterogeneity. *Acta Oncol* **33**: 383–389

Nordsmark M, Overgaard M and Overgaard J (1996) Pretreatment oxygenation predicts radiation response in advanced squamous cell carcinoma of the head and neck. *Radiother Oncol* **41**: 31–39

Rockwell S and Moulder JE (1990) Hypoxic fractions of human tumors xenografted into mice: a review. *Int J Radiat Oncol Biol Phys* **19**: 197–202

Rofstad EK (1994) Orthotopic human melanoma xenograft model systems for studies of tumour angiogenesis, pathophysiology, treatment sensitivity and metastatic pattern. *Br J Cancer* **70**: 804–812

Schwickert G, Walenta S, Sundfør K, Rofstad EK and Mueller-Klieser W (1995) Correlation of high lactate levels in human cervical cancer with incidence of metastasis. *Cancer Res* **55**: 4757–4759

Stone HB, Brown JM, Phillips TL and Sutherland RM (1993) Oxygen in human tumors: correlations between methods of measurement and response to therapy. *Radiat Res* **136**: 422–434

Teicher BA (1994) Hypoxia and drug resistance. *Cancer Metastasis Rev* **13**: 139–168

Vaupel P, Kallinowski F and Okunieff P (1989) Blood flow, oxygen and nutrient supply, and metabolic microenvironment of human tumors: a review. *Cancer Res* **49**: 6449–6465

Vaupel P, Schlenger K, Knoop C and Höckel M (1991) Oxygenation of human tumors: evaluation of tissue oxygen distribution in breast cancers by computerized $O_2$ tension measurements. *Cancer Res* **51**: 3316–3322

Walenta S, Salameh A, Lyng H, Evensen JF, Mitze M, Rofstad EK and Mueller-Klieser W (1997) Correlation of high lactate levels in head and neck tumors with incidence of metastasis. *Am J Pathol* **150**: 409–415

Wilson DF and Cerniglia GJ (1992) Localization of tumors and evaluation of their state of oxygenation by phosphorescence imaging. *Cancer Res* **52**: 3988–3993

Wong RKW, Fyles A, Milosevic M, Pintilie M and Hill RP (1997) Heterogeneity of polarographic oxygen tension measurements in cervix cancer: an evaluation of within and between tumor variability, probe position, and track depth. *Int J Radiat Oncol Biol Phys* **39**: 405–412

© Cancer Research Campaign 1998